# A mixed-methods systematic review of the prevalence, reasons, associated harms and risk-reduction interventions of over-the-counter (OTC) medicines misuse, abuse and dependence in adults

**DOI:** 10.1186/s40545-021-00350-7

**Published:** 2021-09-13

**Authors:** Mohammad Algarni, Muhammad Abdul Hadi, Asma Yahyouche, Sajid Mahmood, Zahraa Jalal

**Affiliations:** School of Pharmacy, Institute of Clinical Sciences, College of Medical and Dental Sciences (CMDS), University of Birmingham, Birmingham, UK

**Keywords:** Over-the-counter, Medicines, Misuse, Abuse, Dependence

## Abstract

**Background:**

Over-the-counter (OTC) medicines are typically safe. However, there is evidence that OTC medicines can sometimes cause harm as a result of their misuse, abuse and dependence.

**Aim of the review:**

To review the literature on OTC medicines misuse, abuse and dependence in adults and identify the implicated medicines, contributing factors, associated harms and risk-mitigating interventions.

**Methods:**

Following PRISMA guidelines, electronic databases including Cumulative Index to Nursing and Allied Health Literature (CINAHL), EMBASE, MEDLINE, PsycINFO Web of Science and Google Scholar were searched for peer-reviewed journal articles published in English between January 2011 and March 2019. Quantitative, qualitative and mixed-methods studies assessing aspects of misuse, abuse and dependence of OTC medicines in individuals aged 18 years or more were included. Studies that solely focused on adolescents only, doping in sports or abuse of OTC medicines in people who are substance abusers were excluded. The random effect meta-analysis model was used to pool the prevalence among the population-based studies.

**Results:**

Of 2355 peer-reviewed studies initially identified, 53 were included in this review. According to the study design, the prevalence varied, but the overall pooled prevalence in the population-based studies was: 16.2% for misuse, 2.0% for abuse, and 7.2% for dependence. The common OTC medicines groups involved in the problematic use were analgesics (with or without codeine), sedative antihistamines, cough mixtures containing dextromethorphan. Physical, psychological, social and financial harms were associated with problematic use of OTC medicines in addition to hospitalisation and death. Interventions for the affected individuals were provided mainly through the community pharmacies, general practices and specialised addiction centres.

**Conclusion:**

The problematic use of OTC medicines is quite prevalent in adults, necessitating raising public awareness about their safe use. In addition, innovative harm minimisation models need to be developed, evaluated and implemented across health care settings.

**Supplementary Information:**

The online version contains supplementary material available at 10.1186/s40545-021-00350-7.

## Background

Over-the-counter medicines (OTC), also known as non-prescription medicines (NPMs), are medicines that can be obtained or supplied without a prescription from registered medical practitioners. OTC medicines are frequently used to manage various minor ailments. They are conveniently obtained from community pharmacies, and other retail outlets such as petrol stations, supermarkets and are now increasingly purchased on the internet [1]. OTC medicines promote self-care, benefiting both individuals and the health care systems by reducing the burden on other health care settings [2]. In the UK, it was estimated that the consultations for minor ailment, which can be properly managed in community pharmacy, accounts for 13% of consultations in in general practice and 5% of consultations in Accident and Emergency (A&E) department [3]. However, OTC medicines are powerful pharmacological agents, and the improper use of these medicines in self-medication can lead to patient harm. Some OTC medicines are liable to misuse and abuse, whereas stimulants, laxatives, sedatives, dissociative substances, opiate-containing medicines such as codeine and stop smoking products containing nicotine are the common implicated medicines [4]. The terms 'misuse' and 'abuse' are frequently used to describe the problematic use of OTC medicines reciprocally, but each term has a definite meaning. Previous literature defined misuse as using the OTC medicine for a legitimate medical reason, but improperly, such as taking a higher dose than recommended or using it for a longer duration. On the other hand, abuse is known as using the medicine for an illegitimate medical reason, such as achieving a mind-altering effect or losing weight [5–7]. Dependence and addiction have also been defined as the frequent use of the medicine with the desire to continue using it regardless of its harm and the struggle to voluntary quitting or changing its use [8]. Of note, misusing OTC medicines such as opiates can progress to dependence as a result of both legitimate (misuse) and illegitimate (abuse) purposes [9]. Also, an association have been reported in some individuals between the abuse of OTC medicines and the use of illicit substances [10]. The evidence shows that the problematic use of OTC medicines can lead to harms that range from physical, psychological to socioeconomic harm to the users and their families [4]. The physical harms, for instance, of long-term misuse of codeine-based analgesics containing ibuprofen and paracetamol, involved chronic headache, gastrointestinal haemorrhage, nephrotoxicity and hypokalaemia [11, 12]. Aiming to reduce the risk of OTC medicines' problematic use, several strategies have been adopted, such as raising public awareness, rescheduling medicines, sale restriction, and surveillance [4, 9]. A former review by cooper (2013) investigated the misuse and abuse of OTC medicines, but revealed shortfalls in the following areas; qualitative research aiming to examine individuals' perspectives, estimating the problematic use in a wider range of countries, factors contributing to misuse/abuse, well-evaluated interventions, information concerning the online purchase of OTC medicines, and consensus over defining terms. Therefore, this review aims to provide an updated overview of the extant literature and address questions that have not been addressed yet concerning misuse, abuse and dependence of OTC medicines in adults from 2011 onward [4]. The aims of this review are to (1) report the prevalence of OTC medicines misuse, abuse and dependence; (2) identify classes of OTC medicines implicated in the problematic use; (3) investigate factors contributing to the problematic use; (4) identify harms resulting from the problematic use and (5) identify the adopted interventions to reduce the risk of OTC medicines misuse, abuse and dependence.

## Methods

The current review followed the Preferred Reporting Items for Systematic Reviews and Meta‐Analyses (PRISMA) guidelines [13]. This review's protocol was registered on the International Prospective Register of Systematic Reviews (PROSPERO) with registration number CRD42019127990.

### Databases searched and search strategy

Six electronic databases, including Cumulative Index to Nursing and Allied Health Literature (CINAHL), EMBASE, MEDLINE, PsycINFO, WEB OF SCIENCE and Google Scholar, were searched from January 2011 to March 2019. The search was conducted using an iterative process combining the three search terms "over-the-counter", "medicine", and "misuse". These search terms and synonyms were combined using the Boolean operators OR and AND (Additional file 1: Table S1). The exact terms applied to run the search were customised to the requirements of each database. The searches were only limited to English-language peer-reviewed studies.

### Criteria for including studies in the review

Studies were included in this review if the participants were adults (aged 18 years and more). Studies investigating the problematic use in adolescents only (≤ 18 years) or focused on a special population such as athletes and substance users were excluded. Concerning medicines categories, studies were included if they investigated the problematic use of medicines registered as OTC in the country of the study as OTC medicines categories vary from one country to another. However, studies that examined both prescription and OTC medicines but did separately report data on OTC medicines were included. In terms of study design, all empirical studies (quantitative, qualitative and mixed methods) were included to accomplish the review aims. Examples of quantitative studies could include population-based cross-sectional and cohort studies that estimate the prevalence of OTC medicines misuse, abuse and dependence. Also, interventional studies such as randomised controlled trials, before and after studies which could investigate the effectiveness of interventions in reducing the risk of OTC medicines misuse, abuse, and dependence. Examples of qualitative studies could include those studies employing focus groups or interviews to explore contributing factors, associated harms, help-seeking sources and barriers to interventions from the views of medicines users and healthcare professionals.

### Study selection and data extraction

**Fig. 1:**
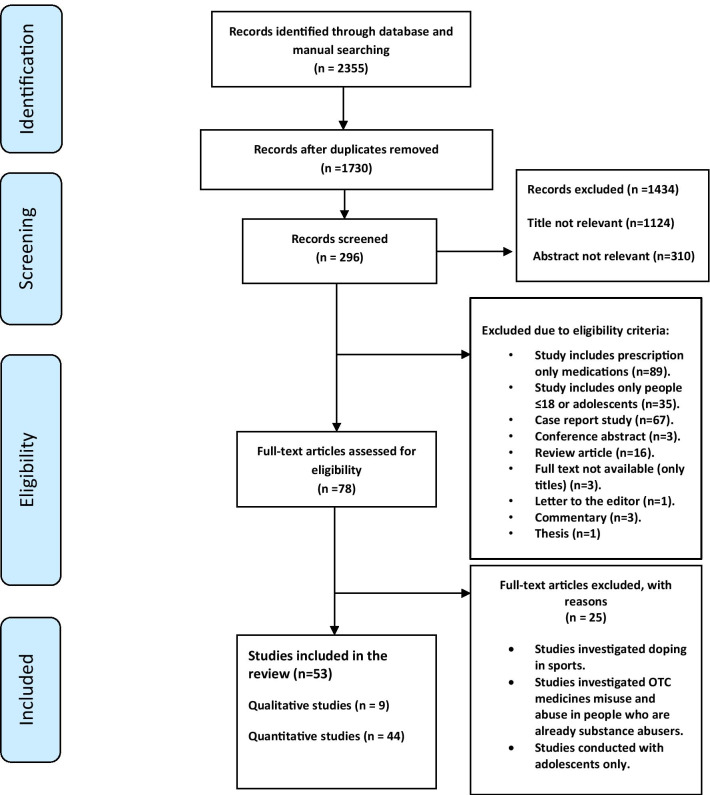
PRISMA diagram of data extraction and study selection process

The selected databases were searched by the first author (MA). The results retrieved from the electronic databases were exported to EndNote X8 and merged, after which duplicates of the same studies were removed. The titles and abstracts were screened to identify potentially relevant studies. The full-text reports of studies that were considered potentially relevant were then obtained and reviewed to check their eligibility. All searching, screening and initial reviewing process were conducted by the principal researcher (MA), and then a second review was conducted by one of the two reviewers (ZJ) and (AY). Disagreements between the reviewers were resolved by discussion. The full-text of the articles that met the inclusion/exclusion criteria was retrieved, and the reason(s) for excluding other studies was clearly indicated (Fig. 1). The following data were then extracted into a customised data extraction sheet from eligible studies: author(s), year of publication, country, aims, design, sample, participants and results (Additional file 1).

### Quality assessment

The Joanna Briggs Institute (JBI) Critical Appraisal Checklists were used to assess the quality of included studies [14]. They are used to appraise the methodological quality and decide the extent to which a study has addressed the probability of bias in design, conduct and analysis. Because this review includes studies with multiple designs, the JBI critical appraisal tools were selected for their widest applicability range and acceptable validity [15]. Checklists for prevalence studies, cross-sectional, case series, cohort studies and qualitative studies were used as appropriate. In addition, the cross-sectional and qualitative research checklists were used to assess the quantitative and qualitative components of the sole mixed-methods study. Studies were categorised as having a high, medium or low risk of bias. Every item of the JBI checklists was answered either yes, no, unclear or not applicable. The percentage was calculated out of the number of positively answered questions to the total number of questions. The quality ranking was as following: low if less than 33% of the items were positively answered; medium if the score was between 33 and 66%, and high if the score was over 66%. This method of scoring was used in a previous systematic review [16]. However, no studies were excluded due to quality assessment to ensure all potential studies can produce a comprehensive picture of research in this area.

### Data synthesis

The review questions were assumed to be addressed by both quantitative and qualitative research studies. Also, the obtained evidence was highly diverse. Therefore, according to the JBI methodology for mixed-methods systematic review, the convergent integrated approach was used to synthesise the evidence [17]. This approach encompassed assembling the qualitative and quantitative data together. Then, assembled data were categorised and pooled together according to their similarity to produce a set of integrated findings. The random effect meta-analysis model was only used to pool the prevalence of misuse, abuse and dependence among the population-based studies.

## Results

The electronic database search yielded 2355 articles, of which 296 articles were considered potentially relevant after title and abstract screening. The full texts of the remaining 78 references were reviewed and evaluated against the eligibility criteria. Finally, 53 articles were included in the review (see Fig. 1).

### Prevalence of OTC medicines misuse, abuse and dependence

Of the 53 included studies, the prevalence of OTC medicines misuse, abuse and dependence were estimated and reported in 14 cross-sectional population-based studies either with pharmacy customers or the general public; one observational walking interview study; four studies examined the perspectives of community pharmacists and five studies retrospectively analysed data reported to national databases. The studies investigated the problematic use with an individual OTC medicine or focused on one or two classes of medicines or investigated the problem with all OTC medicines registered in the country of the study. The prevalence of the problematic use, either 'misuse', 'abuse' or 'dependence', was reported in this review according to the term used in each study.

### Prevalence of OTC medicines problematic use in general public and pharmacy customers

**Fig. 2:**
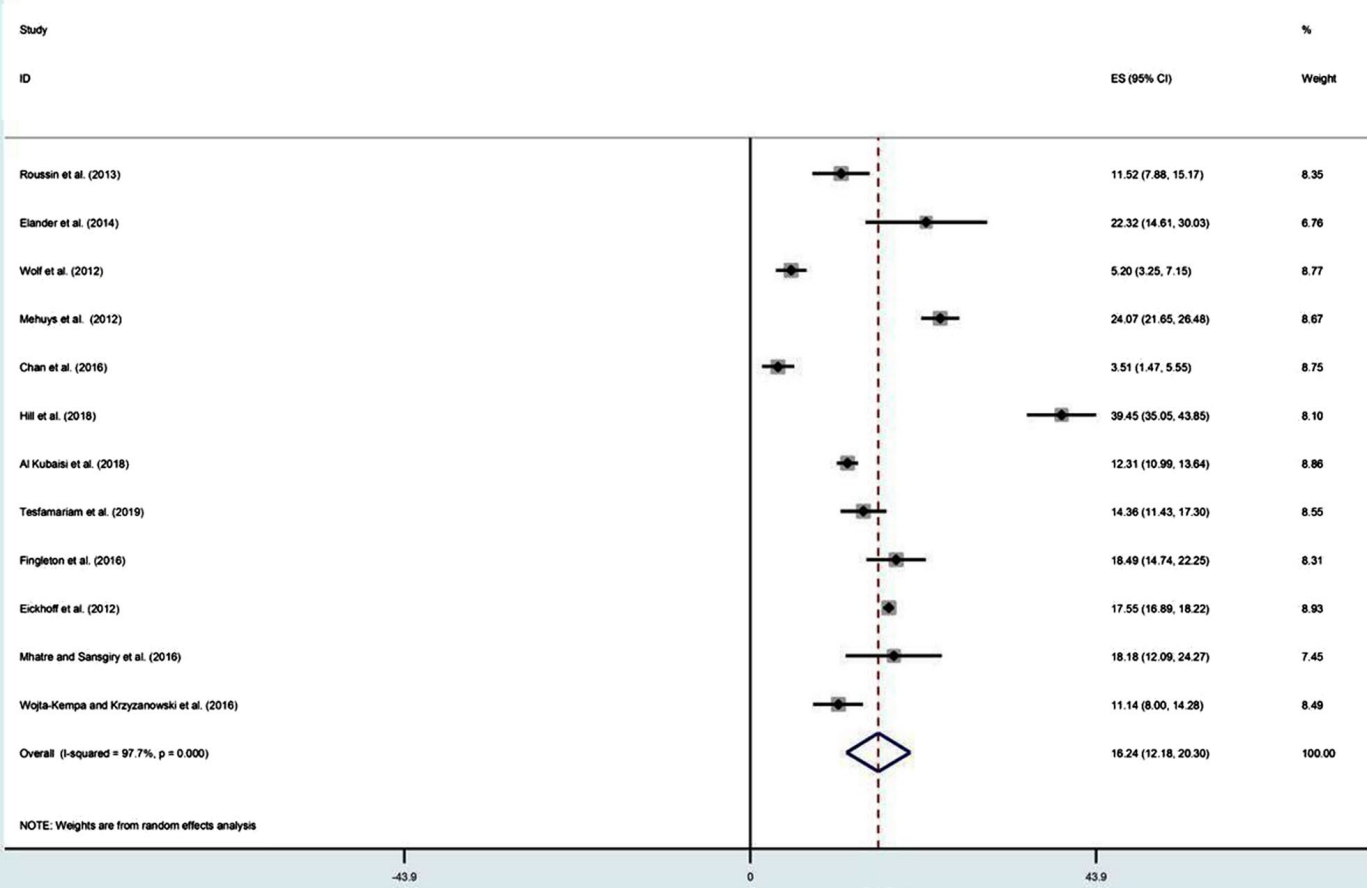
Pooling of prevalence of OTC medicines misuse in population-based studies

**Fig. 3:**
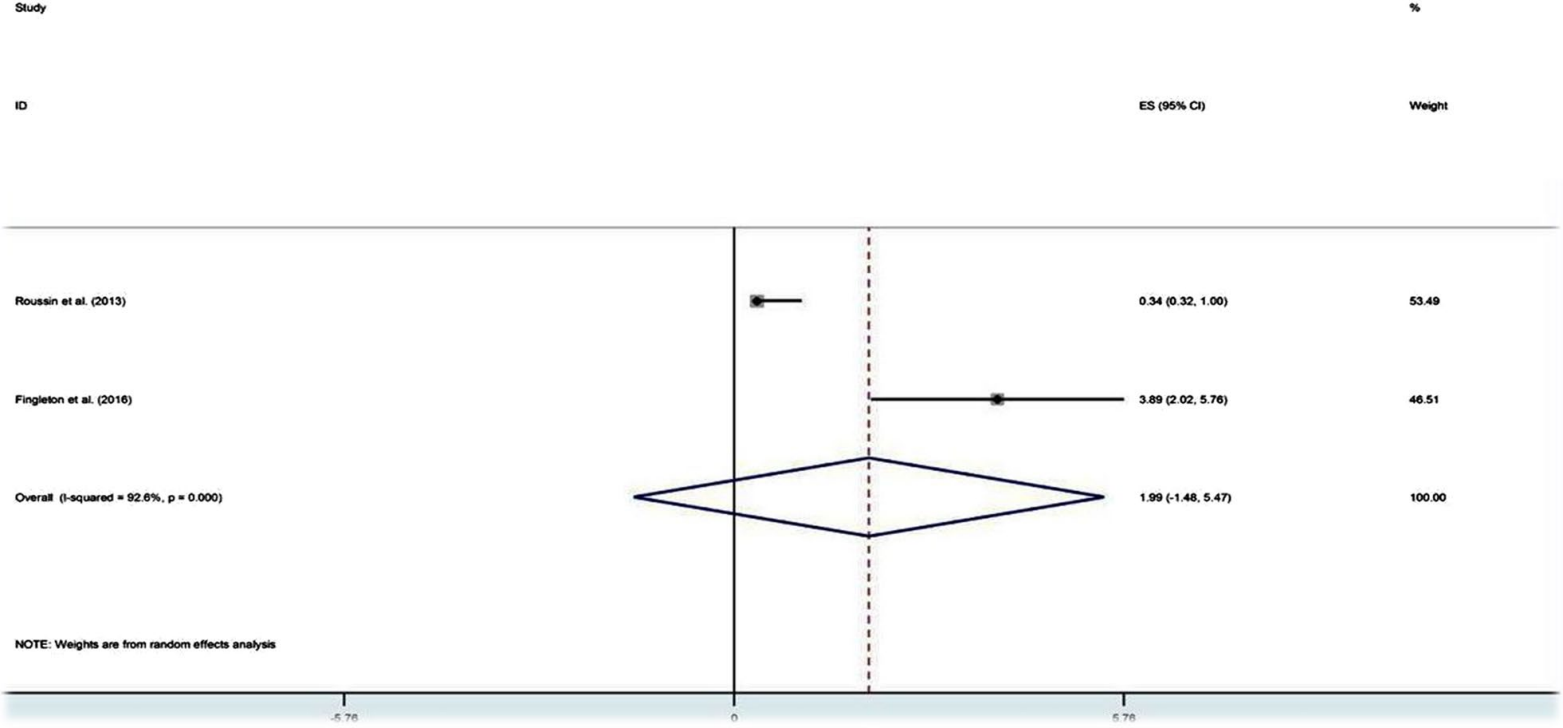
Pooling of prevalence of OTC medicines abuse in population-based studies

**Fig. 4:**
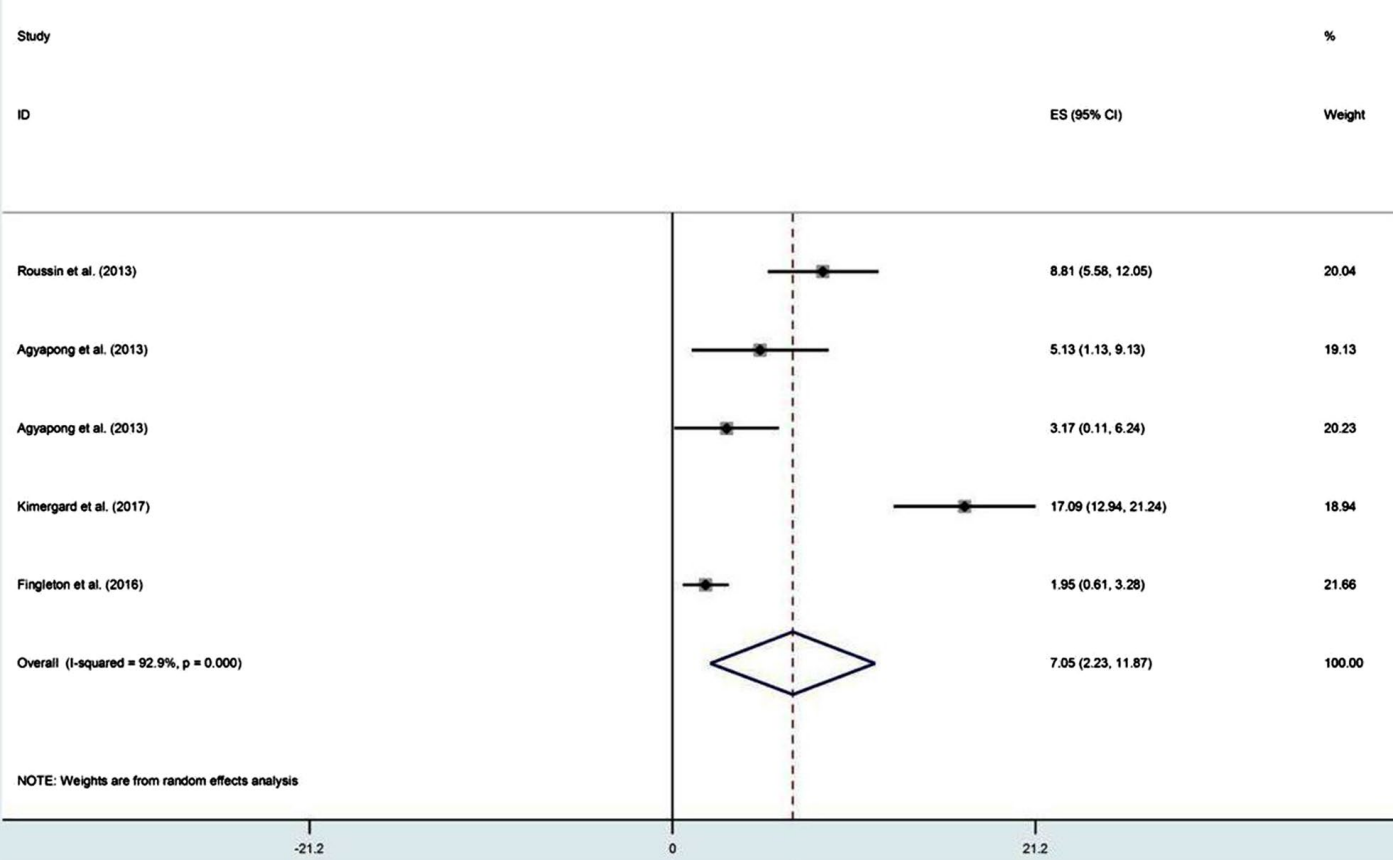
Pooling of prevalence of OTC medicines dependence in population-based studies

Overall, the prevalence reported via survey questionnaires with pharmacy customers and the general public ranged from 3.1 to 59% for misuse, 0.8 to 4.1% for abuse and 4.2 to 17.8% for dependence (see Table1). The pooled prevalence was 16.2% for misuse, 2.0% for abuse and 7.2% for dependence (see Figs. 2, 3 and 4). Among these 14 studies, only two studies, one in France [18] and the other in the UK [19], clearly pre-defined and estimated the prevalence of the three levels of problematic use. In France, the prevalence of misuse, abuse and dependence of codeine-based analgesics was 6.8%, 0.85% and 17.8%, respectively, while the misuse of sedative antihistamine was at a rate of 37% [18]. In the UK, the lifetime prevalence of misuse, abuse and dependence on OTC medicines was 19.3%, 4.1% and 2%, respectively, as misuse and dependence were more frequent with analgesics either containing codeine or not and abuse was more frequent with cold and flu preparations containing sedative antihistamines [19]. Among the gay community in London, the prevalence of misuse of OTC coughs mixtures with sedative properties in the group of males having sex with males was 3.6% versus 3.1% in the group not involving in sexual acts [20]. In Scotland, the prevalence of misuse of OTC analgesics containing codeine and sedative antihistamine in pharmacy customers was 39.45% [21]. Individuals suffering from regular headaches and presenting for self-medication in community pharmacies in Belgium were estimated to misuse analgesics at a total rate of 24%, of whom 58.2% misused caffeine-combined analgesics and 51% misused paracetamol [22]. In the USA, the potential misuse of OTC analgesics containing paracetamol was 5.2% in adult patients waiting for their physicians' appointment at outpatient general medicine clinics [23]. However, the rate was higher (18%) in the elderly consuming different OTC medicines whilst analgesics/antipyretics were the most frequent (50%) used medicines among them [24]. A single observational study employed walking interviews with 20 older adults in a community pharmacy to identify how they hypothetically select an OTC medicine for pain and sleep situations. The study concluded that at least one occasion of potential misuse was detected in 95% of participants, while drug–drug interactions due to OTC sedative antihistamine and analgesics comprise 50% and 60%, respectively [25]. Involving community pharmacists to identify drug-related problems (DRPs) in self-medication with OTC medicines when customers presented symptoms or ordered OTC medicine was considered in a German study [26]. 'Intended duration of drug use too high including abuse' was found in 17.1% as well as a wrong dosage was found in 6.8% of customers. In customers with a history of OTC medicine, significance was found with wrong dosage (*p* < 0.05) and drug–drug interactions (*p* < 0.001) [26].

**Table 1:** Prevalence of OTC medicines misuse, abuse and dependence

Study	Study design	Sample size	Rate (%) of problematic use			OTC medicine
			Misuse	Abuse	Dependence	
Roussin et al. [18]	Cross-sectional	118	6.8%	0.85%	17.8%	Codeine-based analgesic
		70	37.1%	–	–	Sedative antihistamines
Elander et al. [63]	Cross-sectional	112	22%	–	–	Analgesics
Wolf et al. [48]	Cross-sectional	500	5.2%	–	–	Paracetamol
Mehuys et al. [22]	Cross-sectional	1,205	24%	–	–	51% misused paracetamol
						7.2% misused acetylsalicylic acid
						23.6% misused NSAIDs
						58.2% misused caffeine-combined analgesics
Agyapong et al. [39]	Cross-sectional	117	–	–	6.7%	Pre-regulations on codeine supply
		126	–	–	4.2%	Post-regulations imposed on codeine supply
Chan et al. [20]	Cross-sectional	313	3.6%	–	–	Coughs mixtures with sedative properties in MSM (men having sex with men)
			3.1%	–	–	Coughs mixtures with sedative properties in non-MSM
Hill et al. [21]	Cross-sectional	474	39.45%	–	–	60% misused Paracetamol/ Codeine
						14.5% misused Ibuprofen/ Codeine
						14% misused Diphenhydramine
						3% misused Promethazine
Ki mergard et al. [41]	Cross-sectional	316	–	–	17.1%	Codeine-based analgesics
Al Kubaisi et al.[64]	Cross-sectional	2355	22%	–	–	Analgesic/antipyretic (16.5%)
						Anti-allergic (4.9%)
Tesfamariam et al. [47]	Cross-sectional	609	14%	–	–	Analgesics, antipyretics, cough and cold preparations
Fingleton et al. [19]	Cross-sectional	411	19.3%	4.1%	2%	Misuse and dependence were common with OTC analgesics (alone or combined with codeine) and abuse was common with histamine containing products
Eickhoff et al. [26]	Cross-sectional	11,069	Intended duration of drug use too high including drug abuse' was found in 17% and wrong dosage in 6.8%	–	–	OTC analgesics, laxatives and decongestants
Mhatre and Sansgiry. [24]	Cross-sectional	154	18%	–	–	OTC medicines
Wojta-Kempa and Krzyzanowski [37]	Cross-sectional	386	11%	–	–	OTC analgesics
Abood and Wazaify [36]	Cross-sectional	170 (community pharmacists)	57.7% of participants suspected misuse or abuse	–	–	Ketoprofen (11, 3%)
						Chlorpheniramine (5, 7%)
						Codeine-based analgesics (4, 5%)
Wright et al. [54]	Cross-sectional	709 (community pharmacists)	80.8% of pharmacists reported suspected OTC misuse	–	–	Codeine-based products were frequently reported
Weidmann et al. [33]	Cross-sectional	4,026 (community pharmacists)	47.8% of pharmacists agreed that customers did could misuse Orlistat	–	–	Orlistat
Barrett and Costa [34]	Cross-sectional	32 (Community pharmacists)	44%	–	–	Co codamol (codeine-based analgesic)
Cairns et al. [27]	Retrospective	400 (from 2004 to 2015)	19.5% (an average annual percentage change)	–	–	Paracetamol/codeine
			17.9% (AAPC)	–	–	Ibuprofen/codeine
Brass et al. [29]	Retrospective	–	0.0638% (per 1000,000 population per 10 years)	–	–	Paracetamol-combination products
Karami et al. [30]	Retrospective	–	–	Intentional abuse calls form 2000–2015 n = 3472 (11.4 mean rate per million population)	–	Single substance dextromethorphan
Schifano and Chiappini [31]	Retrospective		14.9% (intentional misuse)	0.25%	0.4%	Loperamide
Mill et al. [52]	Retrospective	30	99 admissions for 30 patients due to misuse	–	–	Ibuprofen/codeine
Lee et al. [32]	Retrospective	26	Of cases with known intent (n = 18), 12(67%) were misuse/ abuse	–		Loperamide
Stone et al. [25]	Prospective	20	At least one instance of potential misuse was found in 95% of participants	–	–	OTC pain and sleep medicines

### Prevalence of OTC medicines problematic use reported through national databases

Six studies used retrospective analysis of databases to report the misuse of 4 individual OTC medicines, including codeine, loperamide, dextromethorphan (DXM) and paracetamol. Two studies, one in Australia [27] and the other in Ireland [28] investigated the trend of codeine misuse and assessed the effect of restricting codeine supply on the misuse rate. In Australia, from 2004 to 2015, the average annual percentage change for codeine/paracetamol preparation misuse was 19.5% and for codeine/ibuprofen was 17.9% [27]. In Ireland, The National Poisons Information Centre (NPIC) received 1,851 codeine-containing product poisoning calls from 2005 to 2016, whilst the OTC codeine comprised 70% of these cases [28]. The rate of misuse of paracetamol combination products was found at 0.06% (per 1000,000 population per 10 years) through data reported to the American National Poison Data System (NPDS) from 2007 to 2016 [29]. For dextromethorphan intentional abuse, the NPDS received 3,472 calls (11.4 mean rate per million population) for the period (2000–2015) [30]. Among loperamide-related cases being reported to the European Medicines Agency's (EMA) from 2005 to 2017, the rate of intentional misuse was 14.9%, 0.2% for abuse and 0.4% for dependence, while the rate of misuse/abuse in the USA was 67% of cases with known intent (n = 26) according to data registered on The ToxIC registry from November 2011 to December 2016 [31, 32].

### Perceived prevalence of OTC medicines problematic use from community pharmacists’ perspectives

Four studies examined community pharmacists' experiences toward the extent of OTC medicines misuse in the UK [33, 34], Jordan [35] and Yemen [36]. In the UK, 47.8% of community pharmacists showed that it was likely that customers could and did misuse over-the-counter Orlistat [33], and 44% believed that their customers misuse co-codamol due to frequent purchases [34]. The belief was stronger in Jordan when 88% of pharmacists suspected OTC medicines misuse incidence, while cough and cold preparations followed by systemic nasal decongestants were the highly reported medicines [35]. In Yemen, more than half of the respondents (57.7%) suspected medicines abuse/misuse in their pharmacies, whereas ketoprofen was the most reported misused OTC medicines [36].

### Types of OTC medicines involved in misuse, abuse and dependence

There is an apparent variation across the reviewed studies to quantify the problematic use of OTC medicines as some studies included all available OTC medicines in the country of study. Some included one therapeutic class of medicines, while some focused only on a single OTC medicine. Moreover, few studies investigated the three levels of problematic use with an established definition for each level. Therefore, the most frequently reported misused/abused OTC medicines classes in descending order are analgesics (with or without codeine), sedative antihistamines, cough mixture containing dextromethorphan, antidiarrheal agents (loperamide), decongestants, laxatives and weight reduction agent (Orlistat). Dependence was mainly reported with codeine-based analgesics and with paracetamol and loperamide in only two studies.

### Factors, reasons and circumstances involved in OTC medicines misuse, abuse and dependence

Twenty of the reviewed studies reported reasons and risk factors implicated in misuse, abuse and dependence (Table 2). Acute and chronic pain management was the leading medical reason for individuals misusing OTC analgesics such as paracetamol, nonsteroidal anti-inflammatory drugs (NSAIDs) and other combination analgesics [18, 22, 37]. On the other hand, abuse of OTC medicines for non-medical reasons was frequently reported with codeine-based analgesics, dextromethorphan, sedative antihistamines and loperamide. Individuals who were dependent on codeine-based analgesics and loperamide attributed their dependence to the avoidance of acute opioid withdrawal symptoms and, in some cases, to harm themselves or commit suicide [18, 32, 38–41]. Dextromethorphan was mostly abused for recreational purposes, to commit suicide and obtain a mind-altering effect [19, 42, 43], while sedative antihistamines were abused for improving sleep and relaxation [19, 35, 44]. Other risk factors implicated in OTC medicines problematic use collectively involved behavioural, cultural, socioeconomic and health factors. For instance, socioeconomic factors such as personal and relationship problems, living alone, childhood experiences of a negative divorce process, unemployment and low self-esteem, low educational level and occupation were correlated to dextromethorphan abuse and codeine dependence [40, 42, 45]. Low health status such as impaired visual acuity, long-standing diseases requiring polypharmacy use and advanced age were associated with the increased risk of misusing OTC medicines among the elderly [46]. Moreover, health literacy, low education level, and misunderstanding of OTC medicines' instructions were found correlated to the incidence of misuse in diverse groups, including the elderly, people living in developing and developed countries [46–48]. The indicative behaviours of codeine dependents were doctor/pharmacy shopping and presenting with fake or exaggerated symptoms to obtain a supply of prescription and OTC codeine [41, 49]. The practice of abusers was found different from one country to another. For instance, pharmacists in Jordan experienced that the abusers of OTC antihistamines mix the medicine with drinks (e.g. soft drinks, alcohol) or water-pipes, while in Yemen, the suspected abusers always chew Khat or carry it with them while presenting at the pharmacy. However, in the developed countries, abuse of OTC medicines was associated with illicit drug use and alcohol dependency [11, 35, 36, 50]. Concomitant abuse of OTC medicines with other prescription medicines was reported in some studies [31, 42, 51]. For instance, most individuals who were abusing the OTC loperamide took simultaneously prescription medicines, primarily the antidepressants benzodiazepines, according to the European Medicines Agency [31].

**Table 2:** Factors, reasons and circumstances involved in OTC medicines misuse, abuse and dependence

OTC medicine	Medical and non-medical reasons for OTC medicine misuse/abuse/dependence	Risk factors and circumstances involved in OTC medicine misuse/abuse/dependence	References
OTC medicines (not specified)	Self-management of symptoms Previous medical prescribing/treatment Past use of illicit substances Past alcohol dependency Ongoing medical prescribing/treatment *Reasons for taking overdose:* Severe symptoms The belief that the recommended dose would not be adequate to relieve the symptom The belief that a stronger dose would relieve symptoms faster Previous experience	*Behavioural risk factors* Doctor/pharmacy shopping Internet pharmacy purchases Excessive dosages and extended use Daily use Polypharmacy Suspected misusers and abusers are mostly chewing Khat or carrying it with them while presenting at pharmacies [36] *Miscellaneous risk factors:* Educational level Religion (Muslims were reported more than Christians) [47] Occupation Non-medical college students [64] Knowledge about OTC medicines was significantly associated with risky practice	Cooper [50] Abood and Wazaify [36] Hopkins et al. [49] Tesfamariam et al. [47] Al Kubaisi et al. [64]
Codeine-based analgesics	To manage physical chronic pain (such as arthritis, migraine, or relieve pain following surgical interventions) To manage psychological conditions including depression, anxiety, and stress-related conditions To avoid the symptoms of acute opioid withdrawal To gain a pleasurable sensation Self-harm/suicide To improve sleep To Feel-good effect or to curb craving To treat infections/coughs and colds [39]	*Behavioural risk factors* Doctor/pharmacy shopping Excessive daily dosages and extended use Faking or exaggerating symptoms to get a prescription for codeine Poor social support (such as lone parenting, marital and relationship disharmony, childhood experiences of a negative divorce process, unemployment, and low self-esteem)	Roussin et al. [18], Nielsen et al. [38], Agyapong et al. [39] Van Hout et al. [40] Kimergard et al. [41]
Paracetamol	Persistent use to treat pain (musculoskeletal pain, headache pain, dental pain)	*Behavioural risk factors:* Doctor/pharmacy shopping Excessive dosages and extended use Limited literacy Misunderstanding of OTC acetaminophen product information in community dwelling adults that independently associated with both impaired visual acuity and low literacy skills	Roussin et al.[18] Wolf et al.[48] Hopkins et al.[49] Mullen et al. [46]
Loperamide	To avoid the symptoms of acute opioid withdrawal To gain a pleasurable sensation Self-harm/suicide		Lee et al. [32]
Dextromethorphan	Recreational use Suicide attempt To get mind alerting effect for the believe that it helps women to more likely get conceived as it made secretions more receptive to sperm’ (one respondent) [19]	*Risk factors* Personal problems Living alone and relationship problems Problems at work/school –Curiosity Accidental administration	Koziarska-Rościszewska et al. [42] Pringle et al. [43] Fingleton et al. [19]
Sedative antihistamines	To improve sleep and relaxation To achieve mental-altering effects	*Circumstance in which individuals abuse antihistamine* Mixing the medication with drinks (e.g. soft drinks, alcohol) or with water-pipes (Narghile)	Abraham et al. [44] Wazaify et al. [35] Fingleton et al. [19]
OTC analgesics	To manage tiredness, stressful situations and discomfort To cure hangovers As an alternative for the appropriate medicine	*Risk factors* Sex (female) Age over 55 years Low health status	Wojta-Kempa and Krzyzanowski [37]
Haemorrhoid products	Misused for facial skin care purposes		Fingleton et al. [19]
Sore throat preparations	Misused for its pleasant taste		Fingleton et al. [19]

### Harms associated with OTC medicines misuse, abuse and dependence

Harms resulting from misuse, abuse, and dependence on OTC medicines generally included; physical, psychological, social and financial harm, decreased health-related quality of life (HRQoL), hospitalisation, and death. Harms related to codeine-based analgesics' abuse and dependence were more frequently reported than other OTC medicines in the reviewed studies. Physical harms reported by codeine abusers and dependents were either acute side effects such as urticarial itching, distorted vision and respiratory depression or chronic side effects such as nausea, constipation, liver, bowel kidney failure, anaemia, seizures, ulcers and swollen stomach [18, 51]. Also, codeine dependents reported psychological harms such as depressive mood, anxiety, tiredness, inattention, nervousness and feeling sleepy [18]. Rebound insomnia was reported as a withdrawal symptom by individuals who were dependents on the sedative antihistamine doxylamine [18]. Cough mixtures containing promethazine, ephedrine, pseudoephedrine, codeine, hydrocodone were reported to cause psychotic disorder, schizophrenia, depressive disorder, and dysthymia in abusers admitted for treatment in specialised abuse clinics in Hong Kong [45]. Cardiovascular events such as QTC-prolongation, ventricular dysrhythmias were distinctive of loperamide abuse and dependence in patients presented in hospital with toxicity symptoms [32]. Harms related to dextromethorphan abuse included balance disorders, psychomotor retardation and agitation in individuals admitted to hospital for DXM poising [30, 42]. Social harms were mainly experienced by codeine dependents and commonly include; deteriorated family relationships, inability to continue employment, loss of children, spouses and family homes [51]. Misuse of OTC medicines resulted in decreased health-related quality of life in the elderly population. Significantly, it increased the relevant adverse drug events (ADEs) (*β* = 0.298). As a result, the increased ADEs significantly decreased the patient-reported Physical Component Summary Score (PCS) (*β* =  − 0.312) among the elderly [24]. In one study, misuse of OTC paracetamol contributed to hospitalisation in 5.4% of total exposures and resulted in 51 deaths over ten years observational period [29]. Furthermore, misuse of codeine-based analgesics was attributed to 99 hospitalisations for 30 individual patients with average stay of 5.9 days per admission, of which 10.1% demanded intensive care, and the 99 hospitalisations were estimated to cost the Australian health system AU$1,008,082 in another study [52]. OTC medicines problematic use can contribute to deaths due to suicide overdoses. For instance, 83 suicide overdose cases out of 397 were due to OTC medicines particularly diphenhydramine [53]. In addition, codeine-based analgesics, loperamide and dextromethorphan, were also reported to cause death to individuals who intentionally consumed high doses [30, 31, 49].

### Interventions to reduce the risk and manage OTC medicines misuse, abuse and dependence

Interventions to reduce OTC medicines' problematic use varied across countries and were examined through the experiences of help-seeking misusers, community pharmacists, general practitioners and specialists in addiction centres. Intervention effectiveness such as reclassification of codeine-containing analgesics on problematic use was evaluated in two studies. Community pharmacists reported various actions that are taken with OTC medicines misuse/abuse cases. Such actions include physician referral, shifting to a more convenient medicine, monitoring and registering sales, denying sales, limiting sales, providing restricted pack size, storing products out of customers' sight and increasing vigilance [26, 34, 54]. In Ireland, guidance for pharmacists on codeine supply was issued in 2010. It provided pharmacists with criteria to be followed when selling codeine, such as supervision of pharmacists on sales and restricting the supply to three days only before a required medical review. This procedure resulted in a 62% reduction in poisonings of codeine-containing products, including that available as OTC, with a 33% annual reduction from 2010 to 2011[28]. Moreover, this tight regulation in Ireland showed a reduction in the use and abuse of OTC codeine from 6.7 to 4.2% (*p* value = 0.41) among psychiatric patients admitted to psychiatry hospitals [39]. In contrast to this, OTC products containing codeine were up-scheduled in Australia in 2010 to be 'pharmacist only' medicine, but this procedure failed to stop the increase in the rate of OTC codeine misuse [27]. In the USA, the majority of pharmacists (77%) perceived that making pseudoephedrine a prescription-only medicine would minimise methamphetamine abuse and methamphetamine-associated laboratory incidents, while 56% were supporters of the proposed legislation [55]. In general practices, around 20% of codeine prescribers in Ireland showed confidence in recognising codeine dependence without being notified by patients, and 11.4% agreed to have appropriate screening tools in practice. In comparison, 40% of their counterparts in the UK found it challenging to recognise problematic use of codeine without being notified by the patient and showed a lack of confidence in identifying codeine dependence [56]. Codeine prescribers reported that slow or gradual withdrawal was the most followed procedure to manage codeine dependency in addition to education, patient counselling, prescription restriction, psychosocial management, aftercare and referral to specialist care for complex cases [56–58]. Individuals with OTC medicines dependency in the UK reported attempts to seek help from internet support groups, GPs, specialist NHS drug and alcohol treatment services, private clinics, self-management and narcotics anonymous [50].

## Discussion

### Key findings

To our knowledge, this is the first systematic review and meta-analysis that adopted a mixed-methods design to review the literature on OTC medicines misuse, abuse and dependence in adults. The review identified the prevalence, types of medicines implicated, contributing factors, associated harms and risk-mitigating interventions. Concerning the prevalence of the problematic use, the variability in studies remains evident as many designs were employed, identification methods utilised, and study settings examined. In general, the range of prevalence of problematic use reported from all studies in this review was even broader than that reported in a previous review. According to Copper (2013), the prevalence of misuse generally ranged from 37.2 to 46%, with a prevalence of misuse, abuse and dependence on codeine-based analgesics being 15%, 7.5% and 17%, respectively [4]. However, our review was limited to the adults' population, excluding studies solely investigating the problem in young people or specific groups such as substance users and athletes. The studies that investigated the three levels of the problematic use and clearly defined each level, employed the same design, used the same identification method and targeted the same population gave convergent results. For instance, in France, the prevalence of misuse, abuse and dependence of codeine-based analgesics was 6.8%, 0.85% and 17.8%, While in the UK, the prevalence was 19.3%, 4.1% and 2%, respectively [18, 19]. Such results are not comparable with other studies that pre-defined the problematic use but employed a different study design and targeted a specific population. For instance, Stone et al. (2017) employed a prospective study design involving walking interviews with older adults in a community pharmacy to observe their hypothetical selection process when choosing OTC medicines for sleep and pain scenarios [25]. The study identified a very high potential misuse rate (one occasion of misuse in 95% of participants) in a population who are at high risk due to age and polypharmacy. Although the reported misuse is potential and could be prevented by a pharmacist upon a consultation, employing such a direct observational technique could reduce the subjectivity bias and provide a more accurate estimation of the problematic use. In terms of terminology, various terms were used interchangeably, i.e. 'misuse', 'abuse', 'dependence', 'inappropriate use', 'non-medical use' and 'risky practice'. Consequently, this variability might have led to inaccurate, unreliable, and inconsistent descriptions of the extent of problematic use. Moreover, the interchangeable use of terms made it occasionally difficult to distinguish between therapeutic error, unintentional misuse, and intentional abuse. Therefore, to improve comparability of prevalence estimates, as well as to allow combining information from various studies, standardisation should be considered for the following aspects in the planning of new epidemiological studies in this area of research; the terminology used to describe the problematic use, population under the study, e.g. gender, age range, setting, and the study design variables, e.g. the total number of individuals under study, source of data, method of data collection and period of the data collection.

There are many reasons for the problematic use of OTC medicines. Predominantly, the need to manage frequent headaches and chronic pains were the main reasons for misusing OTC analgesics [18, 22, 26]. Therefore, this supports the need for providing better medical care for these chronic underlying conditions to help prevent the consequences of prolonged use. The findings also stress that older people are at higher risk of misusing OTC medicines than others [24, 25, 46]. Specific factors attributed to this include low visual acuity, low health literacy, and concomitant prescription medicines [24, 25, 46]. As a result, this led to adverse drug events (ADEs) and negatively impacted the health-related quality of life (HRQoL) [24]. The non-medical use of OTC medicines is frequently associated with products comprising codeine, dextromethorphan, antihistamines, and pseudoephedrine. Prolonged use and overdosing of these medicines have resulted in full substance dependence [41]. Moreover, individuals who abuse these medicines may also suffer from poisoning from other active substances present in compound formulations, e.g. paracetamol or ibuprofen. Correspondingly, the poisoning was high in individuals who abused medicines such as DXM to potentiate the effect of alcohol and other illicit substances [42].

Harms resulting from OTC medicines' problematic use are not confined to physical, phycological, and social life consequences to individuals but involve an economic cost to the health care system. For instance, abuse of OTC codeine-containing analgesics, particularly for patients who develop dependence and suffer serious morbidities, resulted in hospitalisation and multiple admissions [52]. Individuals found dependents on codeine-based analgesics were more likely to report higher rates of psychiatric illnesses, prescription medicine use, and history of illicit drug use than non-dependent users who primarily used codeine to manage pain [38–40].

Concerning the interventions adopted to reduce the risk of OTC medicines problematic use, up-scheduling codeine to be pharmacist only medicine was the only intervention in the reviewed studies that investigated the impact on reducing the problematic use. However, the impact was variable when the misuse rate remarkably reduced in Ireland but continued to increase in Australia. Community pharmacists are the only healthcare professionals who interact with customers in self-medication and provision of OTC medicines. Their involvement in consultation concerning OTC medicines use revealed problematic use in one of five encounters. Furthermore, 60% of the encounters were completely resolved in the pharmacy without further referral to physicians [26]. From the studies that identified stakeholders and consumers views toward up-scheduling codeine to be prescription-only medicine, mixed views were perceived by both sides. It is noteworthy that interventions to mitigating codeine problematic use could consider other innovative practices. For instance, pharmaceutical drug formulation technologies are evolving to produce disincentives to minimise the occurrence of product tampering and abuse, such as using physical and chemical barrier techniques for producing abuse-deterrent medicines, which could lower the appeal of manipulating opioids [59–62]. These approaches could also be effective if utilised when producing OTC medicines with potential misuse [59].

### Implications for practice, policy and future research

This review provides several recommendations for policy, practice and future research. The current evidence demonstrates that health literacy, low educational level, lack of knowledge and ignorance of reading the patient information leaflet contribute to the OTC medicines' problematic use. Therefore, public awareness and knowledge should be raised about the safe use targeting people who were identified as the most vulnerable groups in this review, including patients with frequent pain, older people on polypharmacy and chronically ill patients. Pharmacists' supervision on OTC medicines sale should be enhanced through considering behind the counter or pharmacist only class of medicines. Future research may consider implementation and evaluation of integrated harm minimisation model across health care settings. Finally, the extent of OTC medicines' problematic use associated with the online purchase and its monitoring should also be examined.

### Limitations

This systematic review has some limitations. First, several studies may show important results, but were not included in this review as they focused only on specific groups such as substance users and athletes. Second, all studies that met the inclusion criteria were included in this systematic review irrespective of their quality assessment. We considered including all the potential studies concerning OTC medicines problematic use to produce a comprehensive picture of the research in this area. Third, because of the heterogeneous methodological nature and reporting of the data, we were able to only undertake a meta-analytical approach on 14 cross-sectional population-based studies that reported the prevalence of OTC medicines problematic use. Lastly, inconsistency was shown across the results of the included studies due to lack of standardisation, whether in the terminology used to assess each level of the problematic use, type of data collected, and to a lesser extent the type of OTC medicine registered in each country.

## Conclusion

The prevalence of OTC medicines' misuse, abuse and dependence are still significant worldwide. As a result, associated harms varied from physical, psychological, social and financial to a reduced health-related quality of life, hospitalisation and death. Legitimate and illegitimate medical reasons beside behavioural, cultural, and socioeconomic factors were involved in the problematic use. Attempts to mitigate the risks were undertaken by community pharmacists, GPs and specialists in addiction centres. Public knowledge and awareness about OTC medicines' safe use should be enhanced, focusing on individuals at risk. Harm minimisation models, including screening and interventions, should be developed, evaluated and considered for wider implementation in relevant health care settings.

## Supplementary Information

**Additional file 1: Table S1.** The systematic review search terms. **Table S2.** Characteristics of the studies included in the systematic review.

## Data Availability

The authors declare that the data supporting the findings of this study are available within the article.

## Abbreviations

OTC: Over-the-counter medicines
NPMs: Non-prescription medicines
A&E: Accident and emergency
PRISMA: Preferred Reporting Items for Systematic Reviews and Meta‐Analyses guidelines
PROSPERO: International Prospective Register of Systematic Reviews
CINAHL: Cumulative Index to Nursing and Allied Health Literature
JBI: Joanna Briggs Institute
DRPs: Drug-related problems
NPIC: National Poisons Information Centre
NPDS: National Poison Data System
EMA: European Medicines Agency
NSAIDs: Non-steroidal anti-inflammatory drugs
DXM: Dextromethorphan
HRQoL: Health-related quality of life
ADEs: Adverse drug events
PCS: Physical Component Summary Score
