# Supplementary material for: A mixed-methods systematic review of the prevalence, reasons, associated harms and risk-reduction interventions of over-the-counter (OTC) medicines misuse, abuse and dependence in adults
Source: J Pharm Policy Pract. 2021 Sep 13;14:76. doi: 10.1186/s40545-021-00350-7 (PMC8439034; doi:10.1186/s40545-021-00350-7)
Supplement: Supplementary file 1 — Additional file 1: Table S1. The systematic review search terms. Table S2. Characteristics of the studies included in the systematic review. [file 40545_2021_350_MOESM1_ESM.docx]

Table S1: The systematic review search terms

| **Over the Counter search terms** | **Medicine**  **search terms** | **Misuse**  **search terms** |
| --- | --- | --- |
| Over-the-counter OR OTC OR behind-the-counter OR prescri$ OR nonprescri$ OR pharmacy medicine$ OR general sales list. | medicine$ OR medication$ OR drug$ OR agent$ OR product$ OR painkiller$ OR analgesic$ OR codeine$ OR pseudoephedrine$ OR dextromethorphan$ OR antihistamine$ OR laxative$ | misus$ OR abus$ OR dependen$ OR addict$ OR pharmacodependence OR "non-medical use" OR recreational use OR irrational use |

**Table S2: Characteristics of the studies included in the systematic review**

| **Authors** | **Country** | **Study aim** | **Study Design** | **Participants/Sample** | **Results** |
| --- | --- | --- | --- | --- | --- |
| Cooper.  (42) | UK | To explore the experiences and views of people who self –reported OTC medicines abuse, how did they obtain the medicines and the reasons for use and how did they seek treatment and support. | Qualitative interviews. | Individuals self-reporting OTC medicine abuse recruited via two internet-based support groups to serve those abusers (n=25). | Addiction to OTC medicines was associated at first with medical purposes and linked to medical prescribing.  Codeine is the essential medicine abused for non-therapeutic effects in distinct dose ranges, but other medicines also reported.  Present treatment may not be appropriate, based on concealed nature of OTC medicines addiction and perceived differences to other forms of addiction. |
| Cooper.  (72) | UK | To explore the experiences and views of community pharmacy staff regarding current practices and concerns, management and support concerning OTC medicines abuse. | Qualitative interviews. | A sample of pharmacy staff in the UK recruited purposively (n=17; 10 pharmacists, 7 medicines counter assistants). | There is uncertainty among pharmacists on to how to manage OTC medicines abuse. Surveillance and procedures are relied on by pharmacy assistants. The commercial environment of the community pharmacy may pose a barrier to preventing abuse and providing support. |
| Tang et al.  (44) | Hong Kong | To depict the clinical characteristics of cough mixture abusers referred to three substance abuse clinics. | Retrospective case review. | Patients presenting for cough mixture abuse at three substance abuse clinics (n=63). | The mean ± SD age of the group was 34.4 ± 6.2 years; 56 (89%) persons were male; 67% were single. About 75% had a forensic record; of these, 32% had drug- related offences, 13% had non–drug-related offences, 24% had both and data of the remainder (6%) were missing. They initially experimented with illicit substances at a mean ± SD age of 18 ± 5 years and commenced cough mixture abuse at a mean ± SD age of 20 ± 5 years. Around 16% of the participants were vitally co-dependent on ‘Z-drugs’ (including zopiclone or zolpidem) and round 25% revealed a history of dependence on Z-drugs as well as sedative hypnotics (11%), heroin (11%) or methamphetamine (13%). The most prevalent psychiatric diagnoses were substance-motivated psychotic disorder (67%), schizophrenia (19%), depressive disorders (11%) and dysthymia (10%). Dextromethorphan was only found in 6% of the urine samples. The most common detected substances were promethazine (75%), pseudoephedrine (67%), codeine (60%), ephedrine (57%), zopiclone (17%) and hydrocodone (16%). |
| Merrill and Sloan.  (73) | US | To describe drug-related deaths according to ethnicity in Utah between 2005and 2010, based on data from the Utah Violent Death Reporting System. | Retrospective database review. | Reported cases of drug overdose deaths in Utah (n=2,661). | Rates of death related to each drug class (OTC medicines, opiates, illicit drugs, benzodiazepines and antidepressants) were significantly larger in non-Hispanics than Hispanics. OTC medicines were involved in 7.5% (n=121, 0.9 per 100,000) of drug-overdose deaths in non-Hispanics and in 5.6% (n=5, 0.3 per 100,000) of drug-overdose deaths in Hispanics. |
| Zheng et al.  (74) | China. | To review the charts of the inpatients, who are cough mixture dependents and specifically investigate the characteristics of those with hypokalemia. | Retrospective case review. | Inpatients who are cough mixture dependents between January 2009 and December 2011 in Guangzhou Brain Hospital, China (n=34). | All the 34 cough mixture abusers were male. Seven of them (20.6%) were diagnosed with transient or severe hypokalemia. Cough mixture abusers with hypokalemia were significantly older in age than those without hypokalemia. The patients’ mean duration of cough mixture dependence and daily dosage were alike across groups. Hypokalemia in these patients normalized by administration of potassium. |
| Roussin et al.  (22) | France. | To examine the prevalence of misuse, abuse, and dependence on non-prescription psychoactive drugs. | Self-administered survey. | Adults asking for paracetamol (control group), codeine combined with paracetamol in analgesics, or sedative antihistamines from randomly approached community pharmacies (n=383). | Misuse was defined as drug use not in line with the Patient Information Leaflet, abuse as excessive drug use having detrimental consequences and dependence was established depending on questions obtained from the Diagnostic and Statistical Manual of Mental Disorders 4^th^ edition (DSM-IV). Misuse, abuse and dependence to codeine analgesics accounted for 6.8%, 0.85% and 17.8% of the 118 patients used these drugs, respectively, which was significantly higher than for paracetamol. 19.5% had used codeine analgesics daily for more than six months. Headache was the most frequent reason for persistent daily use. Misuse was reported by 37.1% of the 70 clients sought sedative antihistamines. |
| Eickhoff et al.  (48) | Germany | To determine drug-related problems in self-medication (OTC medicines use) identified by community pharmacists at the time the drug is dispensed. | Study of consecutive customer requests. | Convenience sample of community pharmacists who were sought to record 100 consecutive customers presenting symptoms or asking for OTC medicine. (109 pharmacists from 103 community pharmacies documented 11,069 customers with 12,567 requests for self-medication). | In general, there were 12,567 requests for self-medication; drug-related problems were identified in 17.6% (n=2,260) of these requests. Of the 2,206 self-medication requests with a drug-related problem, pharmacists identified a total of 2,666 drug-related problems. 'Intended duration of drug use too high including drug abuse' represented 17% (n=455) of drug-related problems; drug classes commonly involved were analgesics, laxatives and sympathomimetic (decongestants). |
| Nielsen et al.  (35) | Australia | To explore the views of over the counter codeine users and issues behind codeine dependence in the community pharmacy setting. | Qualitative interviews | Convenience sample of Australian over the counter codeine-dependent people (n=20). | Key themes emerged included experience of participants requesting over the counter codeine and participants' interactions with pharmacists. The over counter codeine-dependent participants found it generally easy to access over the counter codeine, describing “standard” questioning, minimal intervention from pharmacists and only occasional rejection to supply. A better appearance and presentation were commonly associated with easy codeine supply. |
| McAvoy et al.  (75) | New Zealand | To describe the characteristics of customers addicted to over the counter codeine analgesics presenting to an Auckland open-access clinic and to compare them with customers admitted to a New Zealand detoxification unit and in the Australian community. | Cross-sectional study of patient admissions. | Customers attending a regional, open-access detoxification clinic covering the Greater Auckland area between 1 January and 31 March 2010 (n=15). | Fifteen customers were identified (8% of all new attendances at the clinic) and compared with 77 similar customers identified in Victoria and five other Australian States, and 7 customers admitted to a New Zealand detoxification unit. Cases in each cohort were consistent with those in previous studies, and seem to be similar to each other both demographically and in terms of the high average tablets consumption (49–65 tablets per day), the serious NSAID adverse drug reactions identified, and the long duration of misuse. Many had a history of alcohol or other drug use and mental health disorder. Of the New Zealand Auckland clinic customers, 66% had recently been hospitalized due to intoxication and/or physical problems linked with their over the counter codeine preparation use. Of the 15 customers identified at the clinic, 47% underwent a community-based detoxification, 27% an inpatient detoxification, 13% self-detoxed and 13% were referred for methadone maintenance treatment. |
| Weidmann et al.  (24) | UK | To explore community pharmacists’ early experiences, views and attitudes with over the counter Orlistat, 9 months post legal re-classification between November 2009 and January 2010. | Self-administered survey. | Randomly chosen registered community pharmacies across Great Britain (n=4,026). | 47.8% agreed that it was likely that customers could, and did, misuse over the counter Orlistat. |
| Elander et al.  (64) | UK | To identify predictors of analgesic (prescribed and non-prescribed combined) dependence among people with pain in the general population. | Online Self-administered survey. | General people who had pain and used analgesics in the last month (n=112). | For over the counter analgesics, 9% reported not using in the last month, 29% using once or twice a month, 18% about once a week, 28% more than once a week, 9% almost every day and 8% every day. The rate of misuse (exceeding recommended doses or using for longer than recommended) was 22% for over the counter analgesics. |
| Tobin et al.  (76) | Australia, New Zealand and UK | To analyses the policy response by Australia's National Drugs and Poisons Schedule Committee and compare it with recommendations by expert advisory committees in New Zealand and the UK. | Analysis of public policy documents of responsible regulatory authorities. | Public documents of medicine regulators and their expert advisory committees. | Expert advisory committees in Australia, New Zealand and the UK described the policy problem of over the counter codeine misuse and harm as minor comparable to total use and responded by restricting availability. Pharmacist supervision was needed at the point-of-sale and pack sizes were reduced to short-term use. |
| Wolf et al.  (65) | US | To determine the prevalence of misunderstanding and potential misuse of over the counter medicines containing paracetamol. | Structured in-person cross sectional interviews. | Adult patients waiting for their physician’s appointment at four outpatient general medicine clinics in Atlanta and Chicago (n=500; 125 per clinic). | Generally, 23.8 % of participants reported they would overdose on a single over the counter paracetamol product by exceeding a dose of 4 grams in a 24-hour period; 5.2 % made serious errors by dosing out more than six grams. Additionally, 45.6 % of adults reported they would overdose by ‘double-dipping’ with two paracetamol-containing products. In multivariable analyses, limited literacy (RR 1.65, 95% CI 1.03–2.66) and heavy paracetamol use in the past six months (RR 1.70, 95 % CI 1.10–2.64) were independently linked with overdosing over the counter products. |
| Mehuys et al.  (54) | Belgium | To examine headache characteristics and medication use of individuals with regular headache presenting for self-medication. | Self-administered survey. | Pharmacy customers requesting an OTC analgesic who reported purchasing the analgesic for headache from 152 randomly chosen community pharmacies (n=1,205). | In general, 292 reported medication overuses, 51% of them overused paracetamol, 7.2% overused acetylsalicylic acid, 23.6% overused NSAIDs and 58.2% overused caffeine-based combination analgesics. |
| Hamer et al.  (77) | Australia | To examine how the up scheduling of over the counter combination analgesics containing codeine has impacted the practice of community pharmacists. | Qualitative interviews. | Convenience sample of community pharmacists in West Gippsland, Latrobe Valley and Bendigo areas of Victoria, Australia (n=11; 9 females, 2 males). | Pharmacists reported that they monitor the supply of over the counter combination analgesics containing codeine by recording sales and to intervene at the time they felt that the medication was being taken too frequently. They perceived a number of challenges concerning the supply of over the counter combination analgesics containing codeine including; supply from other pharmacies, establishing therapeutic need, managing codeine dependent individuals, lacking confidence in discussing misuse with people, being unsure where to refer dependent individuals for assistance and customers resentment towards pharmacist involvement in all sales. People who ask for over the counter combination analgesics containing codeine prone to be stereotyped by pharmacists as either ‘genuine’ or ‘misusers.’ |
| Agyapong et al.  (36) | Ireland | To investigate the effects of new regulations on the use of over the counter codeine-based medicines before and after the regulations came into effect. | Self-administered survey. | Psychiatric patients who admitted to an Irish university teaching hospital before the regulations came into effect and 6 months afterwards (n=117 pre-regulations; n=126 post-regulations). | 66.3% and 61.9% were aware that codeine-containing medicines could be addictive pre- and post-regulation, respectively (not significant). 6.7% and 4.2% regarded themselves as being currently addicted to codeine-containing medicines pre- and post-regulation, respectively (not significant). 13.4% and 15.2% were concerned about the frequency with which they at present use codeine-containing medicines pre- and post-regulation, respectively (not significant). 15.9% and 1.9% took codeine-containing medicines for a “feel-good effect or to suppress craving” in the preceding three months pre- and post-regulation, respectively (p<0.001). |
| Van Hout et al.  (37) | South Africa | To explore the unique individual and collective experiences of trajectories of codeine misuse and dependence in  South Africa. | In depth qualitative interviews. | Purposive sample of adult codeine misusers and dependents  (n = 25). | Nine themes with 63 categories emerged, Findings are illustrated: participant profile and product preferences, motives for use, transitioning to misuse and dependence, pharmacy purchasing and alternative sourcing routes, effects and withdrawal experiences, help-seeking and treatment experiences, and strategies for prevention. |
| Wui Ling et al.  (66) | UK | To investigate the prevalence of OTC/prescription medicine misuse in the South London nightclub community and recognize the sources of supply. | Cross sectional survey. | Individuals visiting nightclubs in South London catering for, but not exclusively for, men who have sex with men (MSM) (n=313). | Prevalence of misuse of OTC coughs mixtures with sedative properties in MSM (3.6%) (n=9) versus non-MSM (3.1%) (n=2).  Sources of OTC and prescription medicines reported by the nightclub attendees were; friends, dealers, primary care doctors, internet purchases, overseas and family respectively. |
| Barrett and Costa.  (25) | UK | To examine the perceptions of community pharmacists on the nature and management of Over-The-Counter co-codamol (paracetamol and codeine combination preparations) misuse and abuse. | Cross sectional survey. | Sixty-five pharmacies in Cornwall and 85 pharmacies in Devon (n=150) in the UK.  (32 pharmacists completed the survey). | 88% of participants reported knowing customers who regularly purchased co-codamol, with 44% purchasing co codamol once or twice a week.  The behaviors pharmacist associated with misuse were frequent to purchase and misinformation provided by the patient during consultation.  Counselling and referral are the main interventions undertaken by pharmacist in such cases. |
| Hill et al.  (67) | Scotland | To identify the prevalence of OTC codeine containing analgesics and sleep aid medications misuse in community pharmacies in three geographical areas of Scotland. | Cross sectional survey. | Pharmacy customers (n=474) were surveyed during visit to 15 community pharmacies (n=15) in Scotland. about misuse of codeine containing analgesics and sleep aid medications. | The most prevalent group identified as females, 41–60 years old and employed. It was frequently reported that the recommended treatment duration is exceeded rather than the maximum recommended daily dose. 187 of the completed forms (39.45%) were indicative of misuse of the medication either because of the amount of medication consumed or the duration of consumption.  Paracetamol/Codeine was being misused by over 60% of the group determined as misusing the medication for longer than recommended, with 20.86% using it continuously while Ibuprofen/Codeine accounts for14.5% and 5% ,  Diphenhydramine for %14% and  11% and Promethazine for 3%  With 100% using it continuously. |
| Lee et al.  (34) | USA | To determine the circumstances, dose, clinical presentations, treatment, and outcomes associated with loperamide use using the nationwide ToxIC registry. | Prospective cohort study. | Cases of patients with loperamide misuse and toxicity registered on The ToxIC registry rom November 2011–December 2016 (n= 26). | The median age was 27 and 54% were male. Of cases with known intent (n=18), 12(67%) were misuse/abuse, 3 (17%) were self-harm/suicide, and  3 (17%) were pediatric exploratory ingestions. motives for misuse involved taking higher doses than recommended (n=7), avoiding withdrawal (n=6), and gaining a pleasurable sensation (n=4). Patients consumed higher doses (>200 mg) presented with cardiovascular effects, including QTc prolongation and ventricular dysrhythmias. |
| Hopkins et al.  (47) | Australia | To identify unintentional deaths associated with use of combination analgesic containing paracetamol, codeine and doxylamine, and describe cases characteristics and identify common factors associated with misuse and mortality of these medicines. | Retrospective case series. | Cases of death associated with codeine combination analgesic and reported to the National Coronial Information System between 2002 and 2012. (n=441 death) | 441 unintentional deaths associated with Paracetamol/Codeine products were identified. Doxylamine was detected in 102 cases (23%). Median age at time of death was 48 and 57% were female. Concomitant use of benzodiazepines, other opioids, psychiatric medications, alcohol and illicit drugs was detected in 79% of cases. Behaviors attributed to drug misuse and identified in 24% of cases include doctor/pharmacy shopping, excessive dosages and extended use. |
| Brass et al.  (31) | USA | To assess the contribution of OTC paracetamol containing  Products to Poison Center exposures and the time trend in these exposures since public attention was brought to their potential risks. | Retrospective study. Data retrieved from the National Poison Data System (NPDS) from 2007 to 2016. | Twelve years or older individuals exposed to paracetamol-containing products. | Therapeutic misuse exposures involving OTC paracetamol-containing products reduced from 8753 in 2007 to 6278 in 2016. The majority of exposures observed in individuals 12–29 years of age. More than one paracetamol-based product was included in 24.8% of exposures. Individuals were hospitalized in 5.4% of exposures and 51 deaths occurred in the 10-year observation period. |
| Foley et al.  (52) | UK | To examine prescribing practitioners’ views on prescribed and OTC codeine use, their ability to identify dependence and their options for treatment in the UK. | Cross sectional survey. | Representative sample of prescribing professionals working in primary care and pain settings (n=300). | A total of 76% of the respondents reported that they routinely ask about patients’ use of OTC codeine medicine, and 71% indicated that they document the use of OTC medicines in the patients’ medical notes. Respondents expressed concern about availability of OTC codeine in pharmacies and on the internet (45.8% vs.64%). The vast majority agreed that patients were not fully aware of the risks of dependence with consumption of OTC codeine medicines (83.8%) and believed them to be safe (86.3%). For managing codeine dependence in primary care, slow or gradual withdrawal was the most popular suggested treatment in addition to education and counselling. |
| Abraham et al.  (41) | USA | To determine the percentage of older adults who used an OTC medication containing diphenhydramine or doxylamine and compare their characteristics with older adults using OTC medication that does not contain these ingredients. | Cross sectional survey. | Individuals aged 65 or older reporting taking at least one OTC product to improve sleep within the past 30 days (n=169 of 1025). They were recruited via the Community Registry of the Pittsburgh Claude D.  Pepper Older Americans Independence Center. | Of the 223 OTC sleep products reported by participants, 115 (52%) contained diphenhydramine or doxylamine. Based on the Beers Criteria, more than half of the participants (59%) were found using a potentially inappropriate OTC medicine containing diphenhydramine or doxylamine to improve sleep within the past 30 days. Participants taking at least one diphenhydramine or doxylamine based medicine were less likely to be aware of any safety risks in taking over-the-counter sleep aid medicines than participants not taking these products (38 vs 49%, p = 0.016). |
| Cairns et al.  (29) | Australia | To estimate Australian trends of codeine misuse over the past 12years and investigate whether trends changed following previous rescheduling endeavor in 2010. | A retrospective  review of calls regarding codeine misuse made to the New South Wales Poisons Information Centre (NSWPIC) from  2004 to 15. | Individuals misused codeine about whom calls were made to NSWPIC (n=400). | The frequency of cases rose significantly from 2004 to 2015, with an average annual percentage change (AAPC) of 19.5% [95% confidence interval (CI) =13.8–25.5% P<0.0001] for paracetamol/codeine and 17.9% (95% CI=7.9–28.9%, P<0.01) for ibuprofen/codeine. No significant change in trend was observed at any time, involving following 2010 rescheduling.  The median age of codeine misusers was 34 and 27 years for paracetamol/codeine and ibuprofen/codeine cases, respectively. |
| Gibbins et al.  (78) | Australia | To explore pharmacists' and other health care professionals' views on strategies for managing misuse and/or dependence of OTC codeine containing analgesics in community pharmacy setting. | Delphi survey  (3 iteration). | Experts within the fields of pharmacy and drug misuse and/or dependence agreed to be on the panel (n=40). | The strategies identified by panel as effective and likely to have the most impact on OTC codeine containing analgesics misuse/  dependence in a community pharmacy setting were:  -Utilization of a national real-time database to monitor product sales to help identification of at-risk individuals (100% effectiveness, rank 1 for impact).  -Development of a referral pathway for management of people whom pharmacists have identified as at risk  (95.2% effectiveness rank 2 for impact).  -Training to improve pharmacist communication with people (95% effectiveness, rank 2 for impact). |
| Kimergård et al.  (38) | UK and Ireland | To describe codeine use, dependence and help-seeking behavior. | Cross sectional survey. | Individuals above 18 years residing in the UK or Ireland who reported taking prescribed or OTC codeine in the last 3 months (n=316). | The mean age of respondents was 35.3 years and 67% were women. Of the 316 respondents, 54 (17.1%) scored ≥5 on the Severity of Dependence Scale indicating codeine dependence.  Codeine dependence was associated with daily use of codeine, faking or exaggerating symptoms to get a prescription for codeine and ‘pharmacy shopping’ (P < 0.01).  Larger number of respondents had sought advice on the Internet (12%) rather than from their general medical  Practitioner (5.4%) when less than 1% had sought advice from a pharmacist. |
| McCoy et al.  (79) | Australia | To investigate attitudes of the Australian codeine consumers, pharmacists and general medical practitioners (GP) towards the proposed up-scheduling of OTC codeine to prescription only medication. | Cross sectional survey. | Participants completed web-based questionnaire  (n=354 codeine consumers; 220 pharmacists; 120 GPs). | Most consumers and pharmacists opposed the up scheduling of codeine while only a third of GPs respondents opposed it.  Consumers, on average, doubt that the proposed up-scheduling would address the intended targets of reducing codeine-related side effects and risk of codeine dependence. Similar to consumers, pharmacists expressed concern around whether codeine restriction would address concerns of associated harm and dependence, as well as the burden regular GP appointments would make in terms of finances for consumers and time for GPs. In contrast, GPs did not support these views.  Logistic regressions showed that among consumers, involvement in paid work or study and reporting pain-based reasons for codeine use were significantly associated with opposition to the proposed up-scheduling of codeine (p= <0.001). For health professionals, the role of a GP was found to significantly predict support in respect of codeine up-scheduling, which remained significant, after controlling for age, gender and work site. |
| Mhatre and Sansgiry.  (55) | USA | To examine Spilker’s quality of life model in the elderly population consuming OTC medications. | Retrospective analysis of data obtained from previously conducted cross sectional survey study. Expert panel assessed the presence/ absence of OTC misuse based on patient reported information on drug use characteristics. HRQoL was evaluated using the Short Form-12v2, which contains a physical component summary score (PCS) and a mental component summary score (MCS). | Elderly people consuming OTC medications in Houston, Texas, USA (n=154). | Analgesics/antipyretics (50%) were the most frequent used OTC medications, followed by  Vitamins (17.3%). The most common ADEs reported by patients were nausea and vomiting (33.3%), stomach pain (27.8%), diarrhea (16.7%) and dizziness (8.3%). Misuse of OTC medicines was found in 18.2%. 22.1% of respondents reported ADE as a result of OTC medications. The mean ± SD score of PCS was 40.6 ± 6.8 and MCS was 46.4 ± 7. Misuse of OTC medications significantly increased ADEs related to OTC medications (β = 0.298) and increased ADEs significantly decreased patient reported PCS (β = −0.312), but not MCS (β = −0.213). OTC medication misuse indirectly reduced PCS and MCS by mediating the effect of an increase in ADE; but the association was not statistically significant. |
| Mullen et al.  (56) | USA | To measure the associations between visual acuity and the risk of misuse of OTC paracetamol products in a sample of community-dwelling adults. | Secondary analysis of data following large cross-sectional study. | English-speaking adults at 4 primary care clinics (n = 500). | Of the 500 participants, 39% had limited literacy, and 54% were classified as having low vision.  Low vision was found independently linked to an increased risk of self-dosing errors (odds ratio [OR], 1.67; 95% confidence interval [CI], 1.25–2.21; p < 0.001) and misunderstanding of concomitant-use risks (OR, 1.41; 95% CI, 1.00–2.00; p = 0.05).  Low literacy was found as an independent risk factor for incorrect dosing (OR, 1.71; 95%  CI, 1.25–2.35; p = 0.001) and unawareness of concomitant use instructions (OR, 4.14; 95% CI, 2.80–6.12; p < 0.001). |
| Wójta-Kempa et al.  (70) | Poland | To investigate the most important predictive factors of the abuse and misuse of over-the-counter pain relievers among adults in Wroclaw (Poland). | Cross sectional survey. | Adult people who are residents of Wroclaw (Poland) (n=386). | 91% of participants reported taking OTC pain relievers and 1.3% of them are daily users. The class of “heavy users” accounts for 11% of participants.  The main risk factors for overusing OTC pain relievers were; sex (female), age over 55 years and the low health status.  Only 50% of participants read the box label regularly and 25% of users admit they have changed recommended doses. Over 60% shortened recommended intervals between doses and 16% used two or more different pain medications containing different ingredients.  The non-medical reasons for taking OTC pain relievers were; tiredness (7%), stressful situations (5.4%), discomfort (18.4%), to cure hangovers (26%), and as alternative for the appropriate medicine (7%). |
| Wazaify et al.  (26) | Jordan | To examine the abuse/misuse of prescription and OTC medications in community pharmacies in Jordan in 2014 and to document any changes that may have aroused in the past 8 years. | Cross sectional survey. | A stratified random sample of community pharmacies in Jordan  (n= 290 questionnaire completed). | Pharmacists identified 727 OTC products as being suspected of abuse. From the OTC medications, cough and cold preparations followed by systemic nasal decongestants were by large the most reported medications of abuse, others in a descending order involved simple analgesics, antihistamines, laxatives, and alcohol.  About 47.5% (n = 138) of the pharmacists reported “mixing” medications with drinks (e.g.,  soft drinks, alcohol) or with water-pipes (Narghile; n =  25, 8.6%) to achieve mental-altering effects. The most frequently reported medication to be mixed was Allerfin (chlorpheniramine; n = 11). |
| Koziarska-Rościszewska et al.  (39) | Poland | To identify the social and psychological features of Dextromethorphan (DXM) users. | Retrospective analysis of patients’ records. | Patients from Lodz province hospitalized for DXM poisoning (n=103). | Recreational DXM poisoning was the essential reason for the admission of the majority of patients (53%) and mainly observed in adolescents and young adults. Suicide attempt was reported in 35% of admitted patients. Other motives reported by patients were; personal problems in 34%, problems at work/school in 3.9%, curiosity in 4.9%, accidental administration in 2.9%, and unknown reason in 2.9%.  The average taken dose ranged from 5 up to 120 pills of 15 mg each. Concomitant use with alcohol was reported in 45% of patients.  Clinical manifestations included mainly balance disorders, psychomotor retardation and agitation. Concerning psychosocial risk factors among genders: living alone and relationship problems were significantly more often in females. |
| Karami et al.  (32) | USA | To characterize existing trends and patterns of calls to poison control centers involving DXM abuse, by demographics, geography, common brands, and medical outcomes. | Retrospective analysis of data from the National Poison Data System (NPDS) which captures data on calls to U.S. poison centers on a near real-time basis from 2000 to 2015 regarding DXM intentional abuse. | DXM cough and cold products intentional abuse exposure in multiple age groups. | The annual rate of single-substance DXM intentional abuse calls tripled from 2000 to 2006 but after that the rate plateaued from 2006 to 2015. The highest abuse call rate was noticed in adolescents14–17 years old but it reduced to 56.3% from 2006 to 2015 (143.8 to 80.9 calls per million populations). The most common medical outcome resulted from  DXM abuse was moderate effects (36.6%), followed by minor (30.6%) and no (8.9%) effects, major effects (1.3%) and death (0.02%). |
| Tesfamariam et al.  (69) | Eritrea | To assess the practice of self-medication, prevalence of risky practice and its associated factors in pharmacy outlets of Asmara, Eritrea. | Cross sectional study. | Pharmacy customers in 20 pharmacy outlets in Asmara (n=609). | Among the 609 customers, 93.7% had practiced self-medication with OTC drugs; of which 81.8% were at risky practice. Educational level (p < 0.0001), religion (p = 0.047), occupation (p = 0.027) and knowledge about OTC medicines (p = 0.019) were significantly associated with risky practice. Respondents with elementary and below educational level were 15 times (AOR = 15.49, CI: 1.97, 121.80) at higher risk compared to those with higher education, and students were almost three times (AOR = 2.96, CI: 1.13, 7.73) at higher risk than governmental employees. Around 14% of the respondents confessed that they had consumed more than the recommended dose and 6.9% had experienced drug related problems following use of OTC medications. |
| Norman et al.  (59) | Ireland | To identify best practices in management of opioid abuse  and dependence, essentially codeine, and innovations to meet challenges surrounding safe and compliant use,  Patient awareness-raising, minimizing health harms and enhancing successful treatment of dependence. | Mixed methods approach included: analysis of data collected from the scoping reviews, interviews with key national stakeholders and a circular email request for information on potential innovations to members of the European Medicine’s Agency. | Conversational interviews with stakeholders; Ireland (n=5), the UK (n=6) and South Africa (n=9). | Best practices and potential innovations were identified under the nine headings: (1) manufacture; (2) product information and public education; (3) responsible prescribing; (4) monitoring and surveillance; (5) dispensing, screening and brief interventions in community pharmacies; (6) safety in the workplace and on the road; (7) internet supply of codeine and online support; (8) treatment of codeine dependence; and (9) learning resources and training for health professionals. |
| Foley et al.  (52) | Ireland | To investigate prescribing professional’s perceptions on risk of dependence, screening and management of prescribed and OTC codeine-based medicines. | Cross sectional survey. | Nationally representative group of prescribing professionals (n=398). | 77% of participants agreed to routinely review patient prescribed codeine. 59% of participants usually asked patients about their use of OTC medicines and 50% of them documented use of OTC codeine in their patients’ medical notes. 93% raised concern about the potential to obtain codeine from multiple sources. 88% indicated that patients did not fully understand the risks of taking OTC medicine containing codeine. Only 21% of participants showed confidence in identifying codeine dependence without being informed by the patient and 11.4% agreed to have appropriate screening methods in practice. |
| Kennedy et al.  (30) | Ireland | To assess codeine poisonings reported to The National Poisons Information Centre (NPIC) before and after the new guidance for pharmacists and identify rates of codeine prescriptions following the introduction of restrictions on supply. | Retrospective analysis of data on codeine poisoning. | Codeine‐related poisonings cases reported between 2005 and 2016 (n=1851). | An annual decline was obvious with a significant 33% reduction from 2010 to 2011 (β2 coefficient for level change, 42.1; 95% CI, −68.1 to −16.0; P = 0.006). After 2011, the declining rate of codeine poisonings plateaued. The reduction in poisonings involving OTC codeine products was 62% with a 33% annual decrease from 2010 to 2011 following the new national guidance to pharmacists on the supply of Codeine in 2010. The national pharmacy claims data showed no change in the reimbursement rate for Co-codamol products after 2010 (Incidence rate ratio 1.04, 95% CI, 0.997‐1.08; P = 0.07). |
| Monson et al.  (80) | USA | To examine Kentucky pharmacists’ opinions regarding the effectiveness of current methamphetamine precursor controls, the proposed legislation to make pseudoephedrine (PSE) a prescription only medicine and the potential impact of such legislation on pharmacy practice and patients. | Cross sectional survey. | Kentucky community pharmacists (n=431). | 77% of pharmacists believe that the proposed legislation to make PSE available by prescription only would be effective in reducing methamphetamine abuse and methamphetamine-related laboratory incidents, with 56.2% showing support for the proposed legislation.  Pharmacists working in chain pharmacies were 3 times more likely to support the legislation than their counterparts in the independent pharmacies. Kentucky region of practice, anticipated impact on time spent on PSE activities, pharmacy profit, methamphetamine abuse, and methamphetamine-related laboratory incidents. are factors affected pharmacists’ views |
| Pringle et al.  (40) | USA | To report information on DXM intake patterns, preferences, and perceptions of DXM from a sample of adult DXM users participating in a DXM-focused online community. | Cross sectional survey. | Members of an online community dedicated to DXM issues (n=60). | All respondents admitted illegal and DXM drug use, starting, on average, at 15.7 and 17.1 years of age, respectively. The majority of respondents reported the following: first heard about DXM online or from a friend, preferred to use DXM alone, ingested substances concurrently with DXM to modify its effects, had not been to an emergency room or arrested because of their DXM use, and used DXM for its dissociative and mind-altering effects. |
| Schifano and Chiappini  (33) | Italy | To assess the loperamide related cases being reported to the European medicines Agency’s (EMA's) EudraVigilance (EV) database. | Retrospective analysis of database. | Cases of loperamide-related misuse/abuse/dependence/withdrawal adverse reactions (ADR) from 2005 to 2017 (n=7895). | 1,983 of 7,895 (25.11%) loperamide-related misuse/abuse/dependence/ withdrawal ADR reports. Majority of cases were categorized as drug use disorder (37.4%) or intentional overdose (25.4%) and Intentional product misuse (14.9%). Loperamide was used alone in 41.9% of cases and with other drugs involving antidepressants benzodiazepines and other OTCs. Cardiovascular ADRs were reported in (1,085/7,895 = 13.7%) when conduction abnormalities and EKG alterations were the most frequent. Death resulted in 305/1,983 (15.34%). |
| Sinyor et al.  (46) | Canada | To identify the medications used by individuals who die from suicide by overdose in Toronto and to determine the correlates of specific categories of substances used. | Retrospective analysis of records. | Records at the Office of the Chief Coroner of Ontario covering suicides by overdose in Toronto from  1998 to 2007 (n=397). | There were 397 documented suicides. Most substances detected were psychotropic prescription medications (n = 245), followed by other prescription medications (n = 143) and OTC medications (n = 83).  In suicides where only one class of substance was detected in lethal amounts, OTC medication (n = 48), opioid analgesics (n = 44), and tricyclic antidepressants (n = 44) were most prevalent.  Diphenhydramine was considered the sole cause of death in 14.4% of cases. |
| Stone et al.  (53) | USA | To identify how older adults, select and hypothetically use OTC medications and if the selected medications would be considered safe for use. | Walking interviews. | Walking interviews with community-dwelling older adults (65+) in a community pharmacy (n=20). | At least one occasion of potential misuse was observed in 95% of interviewees. For sleep medications, drug–drug interactions and drug–age interactions were more common, affecting 50% and 65% of participants respectively while in the pain products selected drug–drug interaction affected 60% of the participants. |
| Abood and Wazaify.  (27) | Yemen | To examine abuse /misuse of prescription and OTC medicines in community pharmacies in Aden city and gather information suspected abusers. | Cross sectional survey. | Community pharmacists (n=170). | Almost half of the respondents (57.7%) suspected drug abuse/misuse in their pharmacies. The majority of suspects of prescription and OTC medicines abuse (64.1%) were either chewing Khat or carrying it while obtaining their medicines from pharmacies. The most commonly suspected OTC medicines to be misused/ abused; Ketoprofen (11, 3%) Chlorpheniramine (5, 7%) and Codeine based analgesics (4, 5%). |
| Mill et al.  (45) | Australia | To identify patients admitted to an Australian tertiary teaching hospital over a 5-year period with sequelae of OTC- combination analgesics containing codeine (CACC) misuse and estimate the costs of identified hospital admissions. | Retrospective case note review. | OTC-CACC-related admissions (n=99). | Most of the Ninety-nine admissions (30 individual patients) were related to gastrointestinal morbidities secondary to ibuprofen/ codeine misuse. Mean length of stay per admission was 5.9 days, with 10.1% of admissions demanding g intensive care. The 99 admissions were estimated to cost the health system AU$1 008 082 with a mean cost per admission of AU$10 183. |
| Van Hout et al.  (43) | Ireland | To explore experiences of codeine misusers and dependents in Ireland following the Pharmaceutical Society of Ireland’s 2010 guidelines for restricted supply of OTC codeine containing products. | Qualitative interviews. | Purposive sample of adult codeine misusers and dependents both actively using, in treatment and in recovery (n = 21). | Findings are presented under the following themes: (1) profile and product preferences; (2) awareness of habit forming use and harm; (3) negotiating pharmacy sales; (4) alternative sourcing routes; (5) the codeine feeling; (6) the daily routine; (7) acute and chronic side effects; (8) social isolation; (9) withdrawal and dependence and (10) help-seeking and treatment experiences. |
| Parry et al.  (51) | South Africa | To explore the perspectives of addiction treatment providers regarding treatment for codeine misuse or dependence. | Cross sectional survey. | Addiction treatment providers affiliated with the South African Community Epidemiology Network on Drug Use (n=15) and the South African Addiction Medicine Society (n=5). | Only two of the participants had been delivered specific training on codeine management among many who received training in pharmacological management of opioid dependence. Almost two-thirds of the treatment settings they worked in provided detoxification, pharmacotherapy, psychosocial treatment and aftercare. Participants reported that over half of their codeine patients entered treatment for intentional misuse for intoxication, and dependence resulting from excessive or long-term use. Barriers to patients entering treatment were seen as denial of having a problem, not being ready for change, mental health problems, stigma, and affordability of treatment. |
| Wright et al.  (49) | Scotland | To identify pharmacists’ perceptions regarding OTC medicines misuse, products involved, and measures taken to address misuse over 20 years. | Cross-sectional survey. | Community pharmacists in Scotland (n=709). | The percentage of pharmacists reporting suspected OTC misuse jumped to 80.8% from 70.8% in 2006. Codeine-based products were most frequently perceived to be misused; when Nytol (diphenhydramine) had been most cited in 2006. Among pharmacists reporting suspected misuse, 91.3% had amended policies, involving refusing sales and referring patients elsewhere. |
| Carney et al.  (81) | Ireland, South Africa and UK | To explore the perspectives of Community pharmacists in three regulatory regimes on issues of customer misuse of OTC and prescribed codeine. | Qualitative focus group. | Six focus groups with community pharmacists in Ireland, United Kingdom, and South Africa (n=45). | Findings reported under the following themes:  -Description of popular codeine-containing products and the need for improved medicine information and warning labels. Issues around legitimate availability of codeine and regulatory status; (1) presence of therapeutic need; (2) difficulties in customer–pharmacist communication; (3) business environments and retail focus were raised. (4) Participants also discussed how they identified customers potentially misusing codeine (5) difficulties in relationships between pharmacists and prescribers. |
| Carney et al.  (82) | Ireland, South Africa and UK | To obtain and analyze the opinions and experience of pharmacy staff regarding codeine misuse. | Cross sectional survey. | Pharmacy staff in Ireland (n=464), United Kingdom (n=129) and South Africa (n=124). | Most of the participants reported combination codeine-containing products as most popular, but significantly more pharmacy staff in South Africa reported codeine-containing cough syrups as most commonly popular  (P < 0.001). Codeine use was significantly more of a public health problem in South Africa than in the other two countries (P = 0.02). There was no difference across countries in the level of codeine misuse reported by pharmacy staff. Professional training and education is desired among pharmacists, with unequivocal findings for the need for greater codeine control (P = 0.002). |
| Al Kubaisi et al.  (68) | United Arab Emirates | To investigate the prevalence and associated risks of self-over dosage with OTC medicines in university students in United Arab Emirates (UAE). | Cross sectional survey. | Students in three randomly selected universities (n=2355). | Only 290/1348 (22%) of the participants reported taking more than the recommended dose of oral OTC medicine in the last three months. Analgesic/antipyretic (223, 16.5%) and anti-allergic (67, 4.9%) medicines were more than other classes of the oral OTC medicines. Justifications for taking overdoses were severe symptoms (6%), the belief that the recommended dose would not be sufficient to relieve the symptom (5%), the belief that a stronger dose would relieve the symptoms faster (11%), and previous experience (4%). Identified risk factors for taking more than the recommended dose were high frequently drug-users of daily use (P < 0.001), students from non-medical colleges P < 0.05) and participants with a poly-pharmacy behavior had higher odds of taking over dosage than single medicine users (P < 0.001). |
| Fingleton et al.  (23) | UK | To investigate the prevalence of self-reported misuse, abuse and dependence to  OTC medicines. | Cross sectional survey. | Individuals above 18 randomly selected from the UK Edited Electoral Register (n= 411). | The lifetime prevalence of OTC medicines misuse was 19.3% and for abuse was 4.1%. Regarding dependence, lifetime prevalence was 2% and with 0.8% currently dependent and 1.3% were dependent in the past.  Misuse was reported with OTC analgesics (alone or combined with codeine),  Abuse was reported with cold and flu products containing sedative antihistamines and dependence was reported with OTC analgesics (alone or combined with codeine), sleep aids and nicotine preparations.  The most common reason for abusing an OTC medicine was for sleep or relaxation purposes with products containing sedative antihistamines. A cough remedy was also used by one respondent for trying to conceive for the believe that it helps women to more likely get conceived as it made secretions more receptive to sperm’. Haemorrhoid products were reportedly used for facial skin care purposes and sore throat preparations were used for its pleasant taste. |
